# Leadership, religiousness, state ownership of an enterprise and unethical pro-organizational behavior: The mediating role of organizational identification

**DOI:** 10.1371/journal.pone.0251465

**Published:** 2021-05-11

**Authors:** Tomasz Gigol

**Affiliations:** Personnel Strategies Unit, Institute of Management, Collegium of Management and Finance, SGH Warsaw School of Economics, Warsaw, Poland; Univerza v Mariboru, SLOVENIA

## Abstract

This study proposes a model in which organizational identification mediates the correlations among state-owned enterprises (SOEs), authentic leadership, Christian religiousness, and unethical pro-organizational behavior (UPB). The proposed theoretical framework is based on moral identity theory, social identity theory, and social exchange theory. We tested the hypothesized model using data (N  =  389) from employees of various companies and industries in Poland. Of the respondents, 49.1% worked in SOEs. The reliability and validity of the measures were established. The correlation coefficients among the analyzed variables were obtained using the bootstrap confidence interval method. To thoroughly examine the causal relationships among the variables, covariance-based structural equation modeling (CB-SEM) was adopted. Path analysis was conducted and used to verify a model in which organizational identification mediated the correlations among state involvement in the ownership of an enterprise, authentic leadership, Christian religiousness, and UPB. State involvement in the ownership of an enterprise, authentic leadership, and Christian religiousness were linked to increased organizational identification, which in turn was linked to the intensification of UPB. With the level of organizational identification controlled, state ownership of an enterprise was linked to lower UPB intensity. Limitations, implications and future research directions are discussed.

## Introduction

Unethical behavior by employees, which may result from a poorly expressed eagerness to act for the benefit of an organization, is a significant issue for management science. Examples of this behavior include selling unwanted financial services to unaware customers [1], deliberately delaying payments to suppliers beyond contractual dates [2] or forging the exhaust emission test results of car engines, as occurred at Volkswagen [3]. In the short term, these behaviors may benefit the organization; however, in the long term, they may lead to serious negative consequences, such as degradation of reputation or compensatory liability [4, 5]. They also produce negative consequences for clients and the social environment of the organization [6]. This type of employee behavior has been called unethical pro-organizational behavior (UPB), which is defined as “actions that are intended to promote the effective functioning of the organization or its members (e.g., leaders) and violate core societal values, mores, laws, or standards of proper conduct” [6].

The construct is based on the theory of social exchange, which sees social relations as a system involving the exchange of goods, similar to economic exchange [7, 8]. Social exchange may involve superiors, coworkers, and an organization or part of it. Employees engage in UPB more if they feel a positive sense of mutuality from an organization [9, 10]. Employees whose contribution to the performance and success of an enterprise is substantial may feel that they can neglect some ethical principles [10]. Another study demonstrated that employees are willing to reciprocate good treatment through engagement in UPB [11–13]. This explains the ambiguous influence of leadership on UPB [14]. Some studies suggest that leadership can decrease employees’ engagement in UPB [15, 16], while others indicate that it contributes to its increase [13, 17]. Moreover, numerous studies have concluded that leadership causes employee engagement in UPB to increase through the moderating influence of various factors, such as the ethical climate [18], continuance commitment [19], work engagement [20], moral disengagement [21], reflective moral attentiveness [22], and organizational identification [23, 24].

The other foundation of the construct of UPB is social identity theory, which assumes that to protect the social hierarchy and their own position in this hierarchy, people usually favor the groups to which they belong and discriminate against other groups [25]. Thus, UPB is strongly contingent upon employees’ identification with an organization [9]. This finding has been confirmed by ample research [26–28]. Frequently, organizational identification is also a mediating factor, such as in the relation between leadership and UPB [23, 24] or between unethical standards upheld in a given environment and UPB [29].

Organizational identification influences UPB through moral disengagement [9, 10]. The moral competence of individuals is also a factor that influences the level of UPB [6]. Furthermore, research results indicate that people with Machiavellian traits are more willing to engage in such behavior [30]. “Machiavellianism is conceptualized as one’s propensity to distrust others, engage in amoral manipulation, seek control over others, and seek status for oneself” [31]. Another important factor that influences UPB is employees’ moral development [6].

In accordance with research by Bryant and Merritt [32], moral identity can decrease employee engagement in unethical behavior. The moral disposition of employees is a moderator of the influence of organizational identification on UPB [6, 23] and of social exchange on UPB [10]. Moral identity exerts a direct influence on the reduction of employee engagement in unethical behavior for the sake of superiors and moderates the influence of identification with an organization or the superior on unethical behavior [14, 33].

An amoral corporate culture is another factor that can increase employees’ propensity to engage in UPB [6]. Employees’ acceptance of unethical organizational practices may occur both consciously and unconsciously through a network of formal and informal relations [34], especially considering that managers and employees of the same organization may have different perceptions of the standards of corporate culture [35]. In the departments of an organization that are driven by egoistic standards, employee engagement in UPB visibly increases [29].

We aim to deepen the understanding of the moral, organizational, and relational conditioning of UPB. Our study contributes significantly to gaining deeper insight into UPB and the factors that influence it in four ways. First, we examine the influence of authentic leadership (AL) on UPB. In several studies on the relationship between leadership and UPB, the most frequently used theory is the theory of ethical leadership. To the best of our knowledge, authentic leadership has been studied only three times in the context of UPB [20, 24, 36]. Authentic leadership is a universally recognized concept that refers to management but is not limited to business [37, 38].

Second, to study the influence of individual morality on UPB, we pay attention to employees’ religiousness. In the context of UPB, we have no knowledge of any previous research on factors connected with religion. Religiousness is a factor that predicts numerous ethical decisions at work [39], especially in the face of ethical conflict [40].

Third, with regard to the influence of organizational culture, we direct our attention to state-owned enterprises (SOEs) as a factor differentiating the phenomena linked to UPB. The staff composition of SOEs has been studied in the context of UPB [10], but their belonging to an SOE has not been studied as a factor that differentiates this population from other respondents. In addition, the majority of studies on SOEs, including studies examining UPB, have been conducted in the context of Chinese organizations. We provide a different perspective.

**Fig 1:**
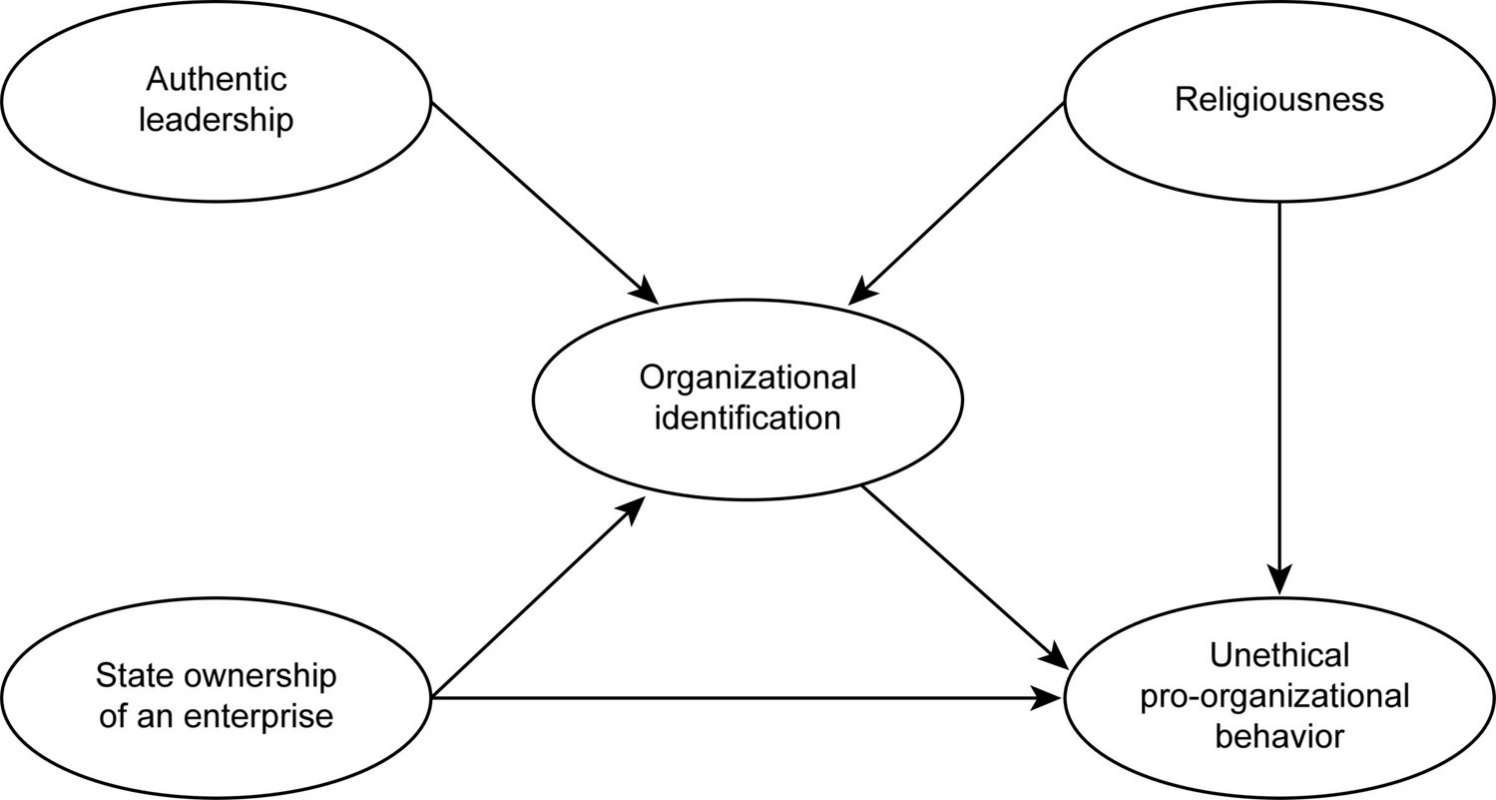
Proposed theoretical framework.

Fourth, a substantial portion of the studies on UPB involves American, Western European, and Chinese research. Central and Eastern Europe are represented by only a few studies. We strive to take a fresh look at UPB from this geographical perspective. Considering the exceptionally high religiousness of Poles, this constitutes an interesting new context for research on UPB. The proposed theoretical framework is presented in Fig 1.

## Theory and hypotheses

### Organizational identification and unethical pro-organizational behavior

Organizational identification is based on the theory of social identity [25]. In this paper, we have adopted Tajfelian definition of social identification proposed by Postmes et al. [41] as a “positive emotional valuation of the relationship between self and ingroup” [41, 42]. Riketta [43] defined organizational identification as “consistency between individual and organizational values”. Organizational identification is also defined as a “perception of oneness with or belongingness to the organization” [44]. Dutton et al. [45] defined organizational identification as a “cognitive link between the definitions of the organization and the self”. Organizational identification causes employees who strongly identify with their workplace to not only follow organizational values but also personally experience the events happening to their employer [44, 46]. Organizational identification has a dark side; for example, if organizational identification is high, overidentification may arise, which leads, among other outcomes, to unethical behavior for the sake of the organization [47]. The influence of organizational identification may also manifest itself in employees’ rationalization of their own unethical decisions [48], especially if these decisions are combined with their conviction of the organization’s readiness to reciprocate [9]. Zhan and Liu [49] demonstrated that superiors evaluated individual results of employees involved in UPB more positively.

Kong [28] noted that passion for work and concentration on work are two factors that determine organizational identification and, consequently, contribute to increased engagement in UPB. The risk of exclusion also increases employee engagement in UPB. Moreover, engagement in such activity increases in situations where an employee has a strong need to be part of the group and there is high risk of being excluded from it [50]. Ostracism also intensifies employees’ engagement in UPB because employees who experience it show greater readiness to perform UPB to ingratiate themselves with the organization [51].

It is worth noting the relationship between the concept of organizational identification and commitment to the organization. Meyer and Allen [52] defined commitment as a psychological state linking an individual to an organization. It is characterized by the following components: affective commitment, continuance commitment, and normative commitment [53]. Affective commitment is an employee’s emotional attachment to an organization and the fact that they identify with it. Continuance commitment is awareness of the costs of leaving an organization, whereas normative commitment is the sense of a moral obligation to stay in an organization.

Organizational identification and commitment to the organization are interrelated and partially overlap because they describe similar mental states [54]. Riketta [43] noted that some researchers use these terms as synonyms [55–57]. It is believed that organizational identification refers to the way a member of an organization defines himself or herself in the context of the organization, while commitment is dependent on the processes of social exchange [58].

Positive influences and phenomena such as organizational identification and organizational commitment may cause some employees to engage in UPB to a greater extent [9]. This phenomenon has been confirmed by numerous studies on UPB, which were cited previously [23, 24]. Therefore, the following hypothesis is proposed:

*Hypothesis 1*. *Employees’ organizational identification influences the intensification of their engagement in unethical pro-organizational behavior*.

### Authentic leadership, organizational identification, and unethical pro-organizational behavior

According to previous research, the influence of leadership on UPB is ambiguous. On the one hand, Wang and Li [16] indicated that moral leadership was associated with a decrease in UPB in followers, and Cheng et al. [15] concluded that responsible leadership, by cascading through mid-level leaders, leads to a decrease in UPB among ordinary workers. However, the findings of Miao et al. [14] demonstrated that there is a curvilinear relationship between ethical leadership and UPB. When the level of ethical leadership increases from low to moderate, the level of UPB increases as well. When, in turn, the level of ethical leadership increases from moderate to high, the level of UPB decreases. Other studies have demonstrated that ethical leadership increases engagement in UPB in employees who hold non-autonomous positions [13].

It is worth noting that there are strong correlations between authentic leadership and ethical leadership [59, 60]. All the above are normative or moral leadership theories [61–63]. With regard to ethics, authentic leadership is the approach that focuses most intensely on the leader’s self-awareness and adherence to his or her own ethical code and refers to external ethical norms or caring about all stakeholders to a lesser extent [64].

The core of the theory of authentic leadership (AL) is the notion of authenticity, which denotes everyday awareness of one’s own experiences as well as taking action consistent with one’s true self and expressing what one actually thinks or believes [65]. The definition of authentic leadership describes four dimensions of authentic leadership: “Leader behavior that draws upon… positive psychological capacities… to foster greater self-awareness, an internalized moral perspective, balanced processing of information, and relational transparency” [66].

Relational transparency involves the readiness of the leader to exchange information with other people, including subordinates, as well as to express his or her authentic “self” in front of others. An internalized moral perspective is concerned with leaders following their internal morality. Those who do adjust their behaviors to align with their values. Balanced processing is related to the leader’s objective analysis of all significant data, including the opinions of subordinates, prior to making a decision. Self-awareness is related to leaders’ understanding of their own strong and weak points as well as the influence they exert on others.

A review of previous research reveals a positive influence of authentic leadership on subordinates’ behavior [67]. In accordance with the GLOBE study, the most common cultural leadership pattern in Eastern Europe is leadership based on values, followed by leaning on the team and orientation toward people [68]. It may thus be stated that authentic leadership is a culturally well-suited concept in the Polish reality, as confirmed by other studies [69].

Authentic leadership positively influences the followers’ identification with an organization because the followers identify with the leader who expresses the objectives and pursuits of the organization [70, 71]. On a group level, authentic leadership also increases employees’ organizational identification through the process of social learning [72, 73]. Moreover, authentic leadership increases employee organizational commitment [74, 75].

Hannah et al. [76] concluded that moral behavior that is not supported by genuine virtue and moral altruism is inauthentic. On the one hand, research findings indicated that authentic leadership is positively correlated with followers’ ethical behavior [77] and prevents subordinates from making unethical decisions when faced with temptation [78]. On the other hand, other studies indicate that if high Machiavellianism is present, the positive correlation between authentic leadership and moral actions is reversed [79]. The ethical component of authentic leadership is not a code of clear-cut ethical norms but rather refers to the virtue of staying loyal to one’s authentic self [64].

Studies in Poland have shown that internalized moral perspective as a component of AL does not have a direct impact on UPB [20]. However, research by Xu et al. [24] demonstrated direct influence of authentic leaders on intensification of UPB among the followers. Niu et al. [36] demonstrated that authentic leadership had the greatest influence on engagement in UPB when it was moderate. The low and high levels of authentic leadership impacted UPB to a limited extent. The result visualization shaped as a reversed U was, thus, similar to the results of research on ethical leadership conducted by Miao et al. [14]. Therefore, it may be concluded that the findings of research on the direct influence of authentic leadership on UPB are unequivocal.

Leadership influences employees’ behavior not only directly but through mediators as well [80]. The results of previous research indicated the influence of authentic leadership on the increase in engagement in UPB through the mediating role of work engagement of employees who hold non-autonomous positions [20]. Within the framework of the concept of authentic leadership, the leader is a role model in an organization and, therefore, is imitated in line with the theory of social learning [64, 73]. It has been demonstrated that authentic leadership has a direct impact on subordinates’ organizational identification [81, 82] and organizational commitment [74, 83]. Organizational identification is a frequent mediator of the influence of the style of leadership on employee behavior [43]. Numerous authors have studied the mediating role of organizational identification in the influence of authentic leadership on, among others, employees’ intrapreneurial behaviors [81], innovative behaviors [84], and individual job performance [85], and new employees’ sense of coping with work [82]. According to research, authentic leadership can increase UPB through organizational identification that additionally reinforces the direct impact of AL on UPB [24]. Likewise, organizational identification turned out to be the mediator of the influence of AL on UPB in the study by Niu et al. [36].

The above deliberations lead us to believe that authentic leadership produces no direct impact on followers’ UPB and to formulate the following hypothesis:

*Hypothesis 2*. *Authentic leadership increases subordinates’ engagement in unethical pro-organizational behavior through the intermediary role of organizational identification*.

### Religiousness, organizational identification, and unethical pro-organizational behavior

Similar to work ethic, religiousness is an important element of human resources [86] and is beginning to be recognized as an element related to organizational management [87]. Religiousness has a certain regulatory power with respect to organizational behavior [88]. This is the case with various religions, such as Islam, Christianity and Judaism [89, 90]. The influence of religiousness on the quality of human capital manifests as work engagement, satisfaction from work, and behavioral ethics [91]. Research by Walker et al. [92] indicated a relationship between one’s religious belief and ethical judgments. On an individual level, a person’s religious beliefs may influence his or her behavior at work. For example, it may be assumed that a person who is dedicated to the Decalogue would be less inclined to engage in unethical behavior at work [93].

Employees’ moral level turned out to be a moderator of the influence of organizational identification on UPB [6, 23]. Moral identity functions to decrease employee engagement in unethical behavior [32]. In turn, religion is a significant source of moral identity [94] and determines the moral identification of ethical decisions made at work [95]. Religiousness or irreligiousness shapes various approaches to business ethics [96] and impacts ethical behavior and ethical decisions [97]. People who find religion important are more ethical than nonbelievers [98, 99]. Furthermore, research findings show that religiousness leads to more ethical behavior by individuals and is a factor that conditions the manner in which decisions are made in the face of ethical conflicts [40, 94, 100]. Ethical decisions inspired by religious beliefs are made in stages: the recognition of a problem, moral judgment, the formation of a moral intention, and the making of a decision [94].

Religious practices and beliefs exert influence on the social and economic life of society [101, 102]. Despite secularization within the youngest generation of the Polish population, the Catholic religion is prevalent in Poland because it was an exceptionally powerful point of reference in social life during communist times [103]. This element distinguishes the Polish within the region. Currently, more than 90% of Polish people declare that they are Catholics [104], and religious minorities are also Christians [105].

Religion enhances one’s readiness to engage in ethical behavior at work due to the conviction that there is a transcendental reality, and it recommends treating ethical behavior as a vocation [106, 107]. Numerous studies indicate that the Protestant work ethic is identical to the Catholic work ethic [108, 109]. Researchers have concluded that Weber’s work ethic, which is historically a religious construct related to Calvinism, is currently universal and completely secular [110]. In Arslan’s [111] research, Muslim managers from Turkey were more attached to the Protestant work ethic than were Protestant managers in Great Britain and Catholic managers in Ireland. However, many authors believe that work ethic is strongly correlated with one’s religion [90].

Religiousness that manifests in convictions and practices is a statistically significant factor predicting one’s response to many ethical decisions [39]. A study with a sample of approximately 1,000 managers in the US indicated a distinct influence of religion on decreased acceptance of ethically dubious business practices [112]. However, many studies do not confirm a relationship between religion and work ethic. Authors of literature reviews regarding the relationship between religion and the ethical decisions of enterprises have not arrived at clear-cut conclusions [113, 114]. In various studies, it has been found that religiousness and participation in religious practices do not significantly differentiate employees or their judgments in terms of business ethics [115]. For example, religion does not result in greater support of corporate social responsibility (CSR) [96, 116]. Taking into account the ambiguity of the previous findings, we propose another research hypothesis:

*Hypothesis 3*. *Christian religiousness decreases engagement in unethical pro-organizational behavior*, *albeit to a small extent*.

Grabowski et al. [117] conducted a study in Poland and concluded that believing that hard work makes logical sense is a variable that predicts UPB. The belief that hard work makes sense is the central element of Weber’s Christian work ethic [109, 118]. Employees’ religiousness influences their normative organizational engagement [119]. The religiousness of assistants in assisted living programs providing social and health care to the elderly notably influences their normative commitment to work [120]. This is consistent with the findings of Weaver and Agle [94] that religious identity has a greater impact on organizational commitment in employees who hold more basic positions and who are not focused on a career.

It is also worth considering spirituality, which is a notion similar to religiousness [121]. de Jager Meezenbroek et al. [122] defined spirituality as “one’s striving for and experience of connection with oneself, connectedness with others and nature and connectedness with the transcendent”. Spirituality, which is understood as a phenomenon that is broader than religiousness, has a strong positive influence on work engagement [123, 124]. Moreover, spirituality influences the organizational commitment of staff [125, 126]. However, based on research in China, Zhang [127] concluded that spirituality increases employees’ engagement in UPB. A strong impact of organizational commitment on employee engagement in UPB has been noted [12, 117]. Organizational commitment is similar to organizational identification [54] and is dependent on the processes of social exchange [58]. Both of these social phenomena constitute the basis of UPB. Therefore, we propose the following hypothesis:

*Hypothesis 4*. *Organizational identification is a mediator between religiousness and unethical pro-organizational behavior and enhances employee engagement in unethical pro-organizational behavior*.

### State-owned enterprises, organizational identification, and unethical pro-organizational behavior

State-owned enterprises are an important part of the world economy and become particularly significant during times of economic crisis. It is estimated that state-owned enterprises produce approximately 10% of the global gross domestic product and more than one-tenth of the largest world companies are state-owned [128]. According to the OECD [129], a state-owned enterprise (SOE) is an enterprise owned and controlled by the central government [130]. However, other authors claim that in a broader sense state-owned enterprises are also the ones that are controlled by the state [131, 132]. Such a definition of a state-owned enterprise has been adopted in this article. Within this understanding, the contribution of state-owned enterprises to the revenues of the enterprise sector in Poland was approximately 13–15% in 2017 [133]. State-owned enterprises have the largest share in the economy in China, where they account for approximately 40% of the GDP [134]. The central government and local authorities directly own 40.31% of the equity of all companies in China [135]. In the remaining countries, state-owned enterprises are usually concentrated around the strategic sectors of the economy, such as the mining, gas, energy, and arms industries as well as postal services.

On the one hand, state-owned enterprises benefit from the close ties with the decision-making centers concerned with the economy; on the other hand, they are less flexible in operation for the same reason [136]. State-owned enterprises are subject to significant protection against competition due to their traditional roles in the economy, relations with the government, and monopolistic or dominant positions in some business sectors [137].

Business ethics differ between state-owned enterprises and the private sector. Research results in China showed that private companies are more prone to illegal activity in response to institutional limitations compared to state-owned enterprises [138, 139]. Ip [140] concluded that there are high ethical standards for business in well-managed state-owned enterprises. Research results indicate that state-ownership in Poland is an important carrier of best CSR (Corporate Social Responsibility) practices and that state-controlled enterprises in Poland are leaders in CSR [141].

Work attitudes and organizational engagement of employees of state-owned enterprises are different than those of employees in the private sector [142, 143]. Employees of state-owned enterprises value work safety and the traditional approach to promotion, training, and recruitment. They also prefer an egalitarian approach to remunerations [144]. In state-owned enterprises, employees are generally rarely dismissed [137] and the employees themselves are unwilling to change their job [142]. Thus, the human resources management practices in state-owned enterprises are more consistent with the hierarchical and traditional approach to human resources management [145].

In Poland, there are major differences in terms of the role of trade unions in private enterprises and SOEs. In private companies, there are often no trade unions, or their significance is very small. In turn, trade unions in SOEs are very important [146, 147]. This is particularly relevant in the sectors of the economy where collective labor agreements have been implemented, especially the energy and mining industries [148]. Strong trade unions in SOEs represent their own interests and are not inclined to engage in the achievement of a company’s goals. The motives for employee engagement in the activities of trade unions are often strongly related to personal benefits and possibilities of having a career in union structures that reach beyond a single enterprise [149]. The strong role of trade unions in SOEs in Poland prompts the conclusion that if employees of SOEs do get involved in any unethical conduct, it is not unethical pro-organizational behavior [136].

Organizational culture influences employees’ unethical pro-organizational behavior [6, 29]. SOE organizational culture is different from the prevalent culture in the private sector. The objectives of state-owned enterprises are frequently linked not only to doing business but also with preserving jobs and the government’s social policy [128]. Hence, unethical pro-organizational behavior should be accepted in SOEs to a smaller extent. For example, organizational commitment in state-owned enterprises more often results in positive behaviors toward other people rather than tasks related to an organization [150]. This is not to say that there is no unethical behavior in SOEs, but its character is different from UPB [151, 152]. Inter alia, the overclaiming happening among employees of SOEs has a significant influence on their ethical behavior [153].

Based on the above deliberations, we put forward the following hypothesis:

*Hypothesis 5*. *State ownership of an enterprise reduces employee engagement in unethical pro-organizational behavior*.

Older and married people as well as the ones who have lived near the workplace for a long time, rather than people from out of town, are more eager to work in state-owned enterprises. Conservative approach to work and social values is also characteristic of the Polish employees of SOEs. Family and religion are still the most important values in

state-owned enterprises other than work ethic, especially in the mining and metallurgical industries [154]. Moreover, employees of state-owned enterprises relied more than others on the company with respect to career advancement [142]. This causes employees of SOEs to identify with their organization to a greater extent than employees of private companies. Employees and the trade unions that represent them are a significant source of influence on state-owned enterprises [136]. This causes greater identification with the organization and thinking in terms of “enterprise is us” [136].

Nevertheless, the character of organizational identification of employees in SOEs is different than in the private sector [142]. Studies have shown that state ownership of an enterprise was a significant moderator of the correlation between stress related to requirements at work and organizational commitment [155]. The staff of state-owned enterprises show greater organizational continuance commitment than employees in the private sector [156]. Comparative examinations of foreign, state-owned, and joint venture enterprises have demonstrated that organizational continuance commitment is higher in state-owned enterprises when affective commitment is comparable [142].

Identification with an organization may bring about both beneficial as well as adverse consequences for a company [48]. On the one hand, organizational identification leads to numerous desirable effects; it reduces staff turnover and increases efficiency as well as job satisfaction [48]. On the other hand, organizational identification may prompt employees to engage in unethical pro-organizational behavior [9, 27]. In particular, employees with low morality engage in UPB through the mediating role of organizational identification [23]. In several studies, it was demonstrated that organizational identification was a mediator between leadership or the unethical norms of the environment and UPB [23, 24, 29]. It was also shown that organizational identification increased the influence of being psychologically demanding on UPB [157] and was a mediator between organizational trust and UPB [158].

Based on the above considerations, we put forward the following hypothesis:

*Hypothesis 6*. *State ownership of an enterprise increases employee engagement in unethical pro-organizational behavior through the mediating role of organizational identification*.

## Materials and methods

### Sample and data collection procedure

The study was carried out within the framework of a research project “Unethical behavior on behalf of an organization” run by the Warsaw School of Economics.

The study was carried out in two rounds. Employees of state-owned enterprises were offered the chance to take part in it. As far as the composition is concerned, this group comprised half of the respondents. The respondents came from large groups of companies from the logistics, power, metallurgy, and mining sectors. The questionnaires from this group were collected in cooperation with company management, ensuring anonymity of the employees. Paper versions of the questionnaire were handed out to the employees. Subsequently, employees put them in a special box. The questionnaires that had been filled out were then handed over to the researchers. The survey was conducted between December 2019 and January 2020.

The remaining respondents represented various enterprises and industries. Thus, 49.1% of the respondents worked in state-owned enterprises, 23.7% of the respondents worked in Polish companies, 22.4% in foreign-owned companies, and 4.1% of the respondents were not aware of the ownership of the company they worked for. The questionnaires in this group were collected during trainings and sessions of postgraduate studies in January 2020. Complete anonymity of the respondents was ensured. Paper versions of the questionnaires were filled out and then collected simultaneously by the researchers. Subsequently, the questionnaires were collected and the results were digitized. The data were subjected to statistical analysis. The relationships between the variables were analyzed using CB-SEM structural modeling methods. IBM SPSS Amos 25.0.0 was used. The estimation was completed on the basis of the highest probability method. The CB-SEM model was applied in order to verify the formulated hypotheses.

Of the respondents, 49.1% worked in SOEs. Employees of firms with Polish capital constituted 23.7% of the respondents, and 22.4% worked in companies with foreign capital. The ownership type of the company was unknown to 4.1% of the respondents. The people under examination worked mainly in medium-sized and large enterprises. Of the respondents, 61.6% worked in companies that employed more than 250 people, and 20.8% worked in companies that employed between 50 and 249 people.

There were 389 respondents in the study, including 204 female (52.4%) and 172 male (44.2%). Thirteen respondents did not provide information on their sex (3.3%). Table 1 presents the frequency distribution of the respondents by age and educational advancement. Members of the largest group of respondents were between 20 and 29 years old. The majority of the respondents had higher education.

**Table 1:** Age and education of the respondents.

Age	*N*	%	Education –	*N*	%
20–29 years	155	39.8	Higher	334	85.9
30–39 years	87	22.4	Secondary	47	12.1
40–49 years	111	28.5	Vocational	3	0.8
50–60 years	18	4.6	No data	5	1.3
60 years and above	3	0.8	Total	389	100
No data	15	3.9			
Total	389	100			

n–number of participants; %–percentage of the sample.

Source: own work based on the research results.

Table 2 presents the frequency distribution of the respondents by job position and period of employment. The largest number of respondents worked as experts. In the majority of cases, the respondents had worked in their company for at least 7 years.

**Table 2:** The respondents’ positions and periods of employment in a company.

Period of employment	*n*	%	Position	*n*	%
Less than a year	82	21.1	Manager/Director	90	23.1
Between 1 and 3 years	103	26.5	Expert/Chief expert	183	47.0
Between 3 and 5 years	36	9.3	Trader	8	2.1
Between 5 and 7 years	28	7.2	Blue collar worker	13	3.3
7 years and longer	133	34.2	Administrative employee	51	13.1
No data	7	1.8	Production employee	2	0.5
Total	389	100	Other	32	8.2
			No data	10	2.6
			Total	389	100

n–number of participants; %–percentage of the sample.

Source: own work based on the research results.

### Measures

#### Unethical pro-organizational behavior

UPB was studied with the unethical pro-organizational behavior questionnaire [9] translated by the authors of the study. We used a 7-point Likert scale from 1 = I absolutely disagree to 7 = I absolutely agree. This scale was previously applied in the Polish context. Factor loadings ranging from 0.72 to 0.84 [117] and 0.66 to 0.81 [20] were obtained.

**Fig 2:**
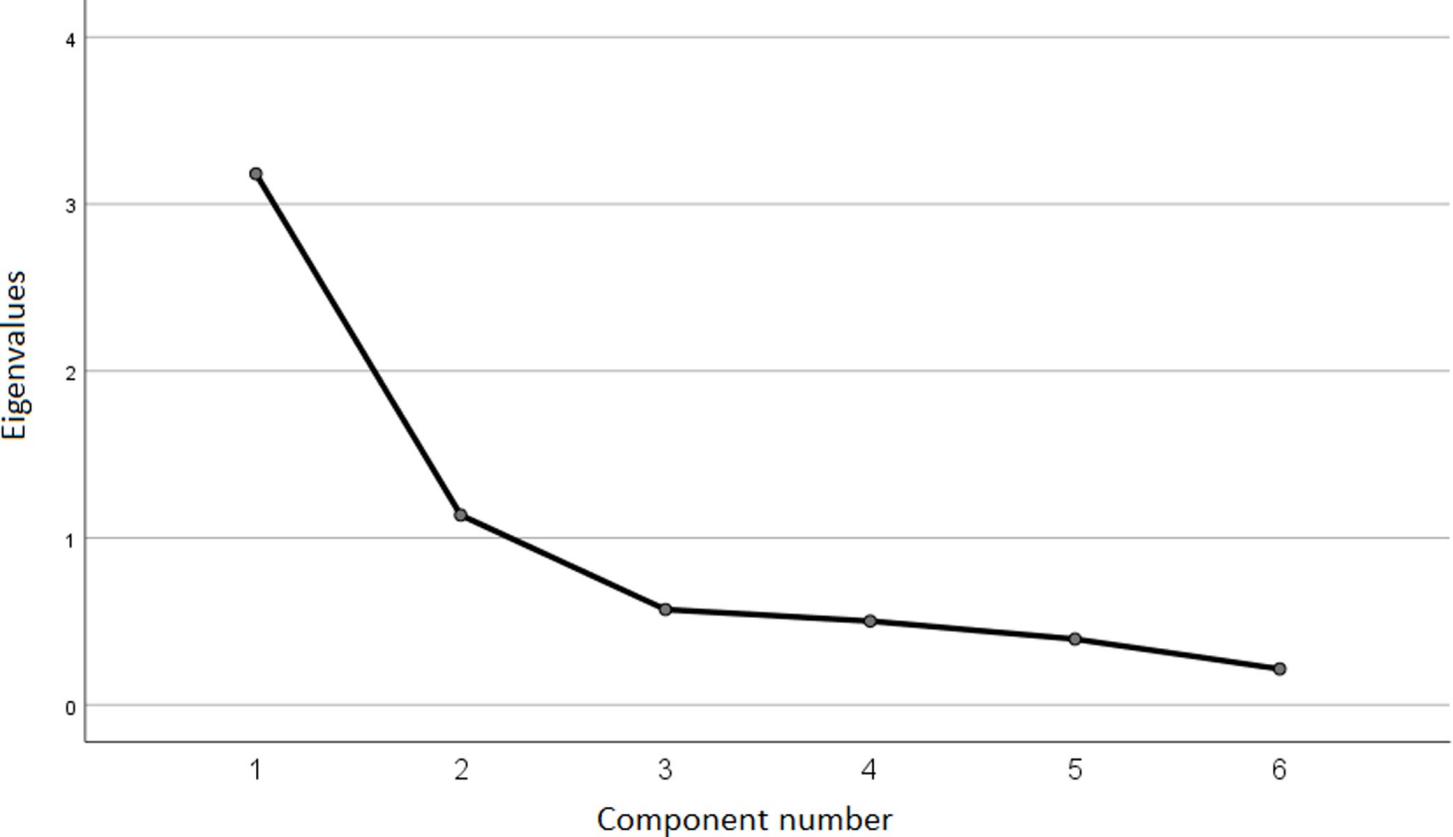
Scree plot obtained via an exploratory factor analysis regarding the dimension of unethical pro-organizational behavior. Source: own work based on the research.

Fig 2 presents a scree plot obtained via an exploratory factor analysis of the *UPB questionnaire* in our study.

The obtained image indicates a unidimensional result accounting for 53.02% of the variance. Table 3 presents the values of the obtained factor loadings for the individual items.

**Table 3:** Values of the factor loadings obtained via an exploratory factor analysis of unethical pro-organizational behavior.

Questionnaire item number	Factor loading
1. If it could help my organization, I would misrepresent the truth to make my organization look good	0.83
2. If it could help my organization, I would exaggerate the truth about my company’s products or services to customers and clients	0.86
3. If it benefited my organization, I would withhold negative information about my company or its products from customers and clients	0.84
4. If my organization needed me to, I would give a good recommendation on behalf of an incompetent employee in the hope that the person would become another organization’s problem instead of my own	0.54
5. If my organization needed me to, I would withhold issuing a refund to a customer or client who was accidentally overcharged	0.41
6. If needed, I would conceal information from the public that could be damaging to my organization	0.76

Source: own work based on the research.

The lowest factor loadings were obtained for items No. 4 and 5. However, factor loadings ^+^/_-_0.40 are considered to meet the minimal level for interpretation of a structure [159]. The required minimum sample must then be 200. In our study, it was N = 389. Loadings of 0.50 or greater are considered practically significant, as in item 4 [159, 160]. The remaining four factors have an impact factor of over 0.7. However, the value of RMSEA fit index of 0.15 was above the 0.1 recommended level.

### Authentic leadership

The study used the Polish version of the *Authentic Leadership Questionnaire* (ALQ) to examine authentic leadership [161]. We used a 5-point Likert scale from 1 = Never to 5 = Very often. The questionnaire has been validated in the Polish context [162]. The factor structure of the Polish version of the ALQ was verified with confirmatory factor analysis based on the maximum likelihood method. The values of the fit index were as follows: *CFI* = 0.95, *RMSEA* = 0.07, and *NFI* = 0.93. These are acceptable levels of the indicators [163]. Table 4 presents the values of the obtained factor loadings. The permission obtained to use the questionnaire in the study does not allow us to publish all of it.

**Table 4:** Values of the factor loadings obtained via a confirmatory factor analysis of authentic leadership.

Dimension	Item number and exemplary statement	
Relational transparency	1. My leader says exactly what he or she means	0.65
	2.	0.82
	3.	0.78
	4.	0.73
	5.	0.58
Internalized moral perspective	6. My leader demonstrates beliefs that are consistent with his or her actions	0.57
	7.	0.64
	8.	0.77
	9.	0.86
Balanced processing	10. My leader solicits views that challenge his or her deeply held positions	0.62
	11.	0.73
	12.	0.77
Self-awareness	13. My leader seeks feedback to improve interactions with others	0.84
	14.	0.71
	15.	0.82
	16.	0.76
Authentic leadership	Relational transparency	0.91
	Internalized moral perspective	0.92
	Balanced processing	1.06
	Self-awareness	0.99

Source: own work based on the research results.

The lowest factor loadings were obtained for items No. 5 (0.58) and No. 6 (0.57). These values allow us to consider the scale to be practically reliable [159, 160].

#### Organizational identification

A single-item social identification measure (SISI) was used to study organizational identification. A 7-point Likert scale was employed, from 1 = I absolutely disagree to 7 = I absolutely agree. It demonstrated high reliability, high validity, and high utility [41, 164–166]. One possible reason for that is the fact that SISI measures the basic concept of organizational identification [41, 42]. It was equally reliable under diverse approaches to and definitions of organizational identification [41], which were mentioned earlier (for more on the topic, see, e.g., [43]). The statement in the questionnaire was “I identify with my company.” The conceptual clarity of the above statement is probably one of the reasons for the infallibility of the SISI [41]. It seems that it is a homogeneous concept [165].

#### Christian religiousness

The Christian Religiousness Scale (CRS) developed in 1988 by Socha [167, 168] was used to study the Christian religiousness of the respondents. The CRS is a Thurstone-type scale [169] and measures attitudes toward the Christian religion regardless of denomination. The items were validated by priests or practitioners in various denominations (Catholic– 12 people, Russian Orthodox– 12 people, Evangelical-Augsburg– 12 people, Adventist –12 people) and research workers (scholars of religious studies– 17 people). The reliability of the scale is very high, and the Kuder-Richardson’s coefficient of internal correlation is rtt = 0.99. The scale contains 20 statements. If a person selects only one statement, the score is equal to the scale value. If a person selects two or three statements, the score is calculated as the arithmetic mean of the summed scale values for these statements [168]. Table 5 presents statements from the Christian Religiousness Scale with the attributed scale values.

**Table 5:** Christian religiousness scale.

Statements	Attributed scale values
1. In reality, religious principles are too rigid.	4.4
2. Faith helps to overcome obstacles.	8.3
3. Faith dignifies people.	8.9
4. People have faith out of fear of death.	3.8
5. Faith is a person’s mainstay.	9.4
6. Religion is God’s love through people’s love.	10.3
7. Faith is a type of demagogical charlatanry.	1.3
8. It is easier to live with religion.	7.9
9. Faith is delusion of the mind.	1.9
10. I am a Christian brought up in religious traditions, but I am not certain of the existence of God.	5.9
11. Religious norms do not let people be themselves.	2.9
12. Faith is the prerequisite of living life to the fullest.	9.8
13. Religion distracts people from the reality of life.	5,1
14. Religion is a pattern of running away from and coming back to God.	7.4
15. Religion does not meet the needs of a contemporary man.	3.4
16. Faith helps and at the same time limits people.	6.5
17. Faith is a cover for a lot of people.	5.5
18. Religion kills people’s creative capacity.	2.5
19. Religion is the meaning of life.	10.4
20. Religion is nonsense of the contemporary world.	1.4

#### Measurement of state ownership of enterprises

In the questionnaire, a question was asked about the significant share of the state in an enterprise ownership structure. In line with the approach adopted by Bałtowski and Kozarzewski [132] as well as Peng et al. [131], we treated the majority and the controlling stake of the state in a company the same way. Responses confirming the significant share of the state were coded as 1 and the remaining responses, that is, private or international significant share in company ownership structure, were coded as 0.

## Results

### Descriptive statistics of the variables under examination

The results were subjected to statistical analysis. Table 6 provides descriptive statistics for the interval variables under analysis. The juxtaposition was supplemented with the results of the Kolmogorov–Smirnov test, which served to verify the presumption of the normal distribution of the variables under examination, and by Cronbach’s alpha reliability coefficients. They turned out to be sufficiently high and reached the level of over 0.7 [160, 170].

**Table 6:** Descriptive statistics of the variables under examination.

	*M*	*SD*	*Min*	*Max*	*Z*	*p*	α
Unethical pro-organizational behavior	3.00	1.17	1.00	7.00	0.06	0.003	0.82
Organizational identification	5.05	1.67	1.00	7.00	0.19	0.001	-
Authentic leadership	3.41	0.85	1.25	5.00	0.07	0.001	0.94
Relational transparency	3.42	0.93	1.20	5.00	0.10	0.001	0.85
Internalized moral perspective	3.50	0.93	1.00	5.00	0.10	0.001	0.83
Balanced processing	3.47	0.98	1.00	5.00	0.10	0.001	0.78
Self-awareness	3.24	0.97	0.75	5.00	0.11	0.001	0.88
Christian religiousness	6.90	2.32	1.30	10.40	0.16	0.001	-

*M*–mean; *SD*–standard deviation; *Min*–minimum value; *Max*–maximum value; α –Cronbach’s alpha reliability coefficient.

Source: own work based on the research results.

Statistically significant deviations from the normal distribution were found for each variable under analysis. All the obtained coefficients of reliability of measurement were appropriately high.

Table 7 presents the r-Pearson’s coefficients of correlation among the variables under analysis, which were obtained with bootstrapping, in line with Hayes’s [171] indications. Statistically significant correlations are highlighted.

**Table 7:** The coefficients of correlation among the variables under analysis obtained via bootstrapping.

	1.	2.	3.	4.	5.	6.	7.	8.
1. Unethical pro-organizational behavior	-							
2. Christian religiousness	-0.157÷0.037	-						
3. Organizational identification	**0.058÷0.268**	**0.030÷0.246**	-					
4. Authentic leadership	-0.125÷0.089	-0.106÷0.108	**0.306÷0.487**	-				
*5*. *Relational transparency*	-0.156÷0.050	-0.107÷0.122	**0.226÷0.413**	**0.884÷0.922**	-			
*6*. *Internalized moral perspective*	-0.150÷0.058	-0.094÷0.114	**0.298÷0.483**	**0.816÷0.898**	**0.669÷0.789**	-		
*7*. *Balanced processing*	-0.059÷0.143	-0.093÷0.111	**0.290÷0.484**	**0.861÷0.903**	**0.635÷0.739**	**0.649÷0.760**	-	
*8*. *Self-awareness*	-0.082÷0.119	-0.132÷0.082	**0.234÷0.442**	**0.880÷0.919**	**0.670÷0.774**	**0.593÷0.731**	**0.778÷0.850**	-
9. State ownership of an enterprise	-0.191÷0.006	**0.023÷0.232**	**0.222÷0.406**	-0.052÷0.159	-0.082÷0.118	-0.031÷0.168	-0.075÷0.130	-0.020÷0.189

Statistically significant correlations are in bold font.

Source: own work based on the research results.

We proceeded to examine the effects of mediation in accordance with Baron and Kenny’s [172] approach. We established that the independent variables are statistically significantly correlated with the mediator. Organizational identification was positively correlated with all the other variables: UPB, Christian religiousness, state ownership of enterprises, and authentic leadership and all its elements (Table 7). Subsequently, we analyzed the correlations between the independent variables and the dependent variable. There was no statistically significant correlation between Christian religiousness and UPB. Thus, at this stage of the study it seemed that hypothesis 3 had not been confirmed. There was no statistically significant correlation between state ownership of an enterprise and UPB. At this stage of the study, it also seemed that hypothesis 5 had not been confirmed by the test results (Table 7). We also noted that the mediator was correlated with the dependent variable in a statistically significant manner. Organizational identification was correlated with UPB at the following level: 0.058÷0.268 (see Table 7). We have been surprised by an outcome indicating that Christian religiousness was found to be correlated with state ownership of enterprises. In addition, all the elements of authentic leadership were correlated with one another.

### Organizational identification as a mediator of the correlations among state ownership of an enterprise, authentic leadership, Christian religiousness, and unethical pro-organizational behavior

Analysis of the coefficients of correlation among the variables under analysis, which are provided in Table 7, produced results that only partially confirmed the proposed hypotheses. In particular, no statistically significant correlations were found between Christian religiousness and UPB, which was the essence of hypothesis 3. Similarly, analysis of the correlations among the variables did not reveal any significant correlations between state ownership of an enterprise and UPB, which was expressed in hypothesis 5.

In social studies, variables may take the form of moderators and mediators [171, 173]. Alluding to MacKinnon et al. [174] and MacKinnon and Fairchild [175], we proposed a research model with a mediator being organizational identification, which is deeply rooted in the relevant literature [43, 66, 81, 82, 84, 85]. Thus, our research sample N = 389 was sufficiently large to exceed the upper quartile of sample size for mediation research, which was 352 [176]. The model also comprised one-way correlations between variables [177].

**Fig 3:**
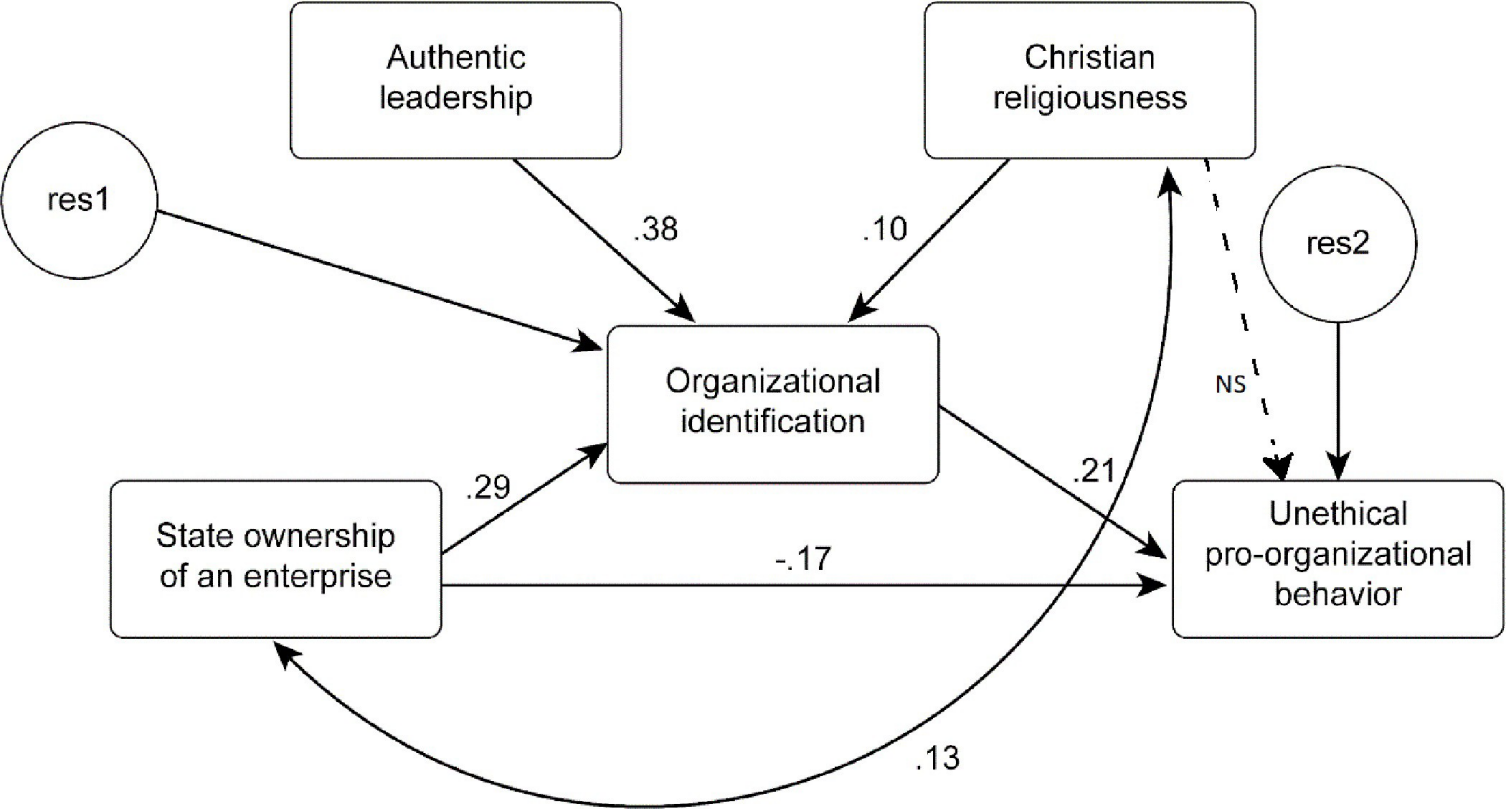
The obtained model of correlations among the variables. Source: own work based on the research results.

To thoroughly examine the causal relationships among the variables, covariance-based structural equation modelling (CB-SEM) was adopted. This method takes into consideration not only the direct correlations between variables but also the indirect and combined ones [178]. The SEM approach facilitates the linear relationship analysis between the latent constructs and manifest variables [179, 180]. We chose this method because the research model we proposed is complex and contains additional covariations of independent variables [181, 182]. Selection of the SEM method was also possible due to the large size of the research sample [183]. Kline [184] suggested N = 200 to be the minimum size of a research sample when conducting SEM analysis. The method increases the reliability of measurement within the framework of research hypothesis testing and simultaneously tests multiple hypotheses [185]. Hence, path analysis was conducted to verify a model in which organizational identification served to mediate the correlations among state ownership of an enterprise, authentic leadership, Christian religiousness, and UPB. The model under analysis was sufficiently well-fitted to the data under examination. The values of the modification indexes were CFI = 0.97 and RMSEA = 0.05. The final model of the correlations among the variables along with the values of the regression coefficients are presented in Fig 3.

It was concluded that state ownership of an enterprise, authentic leadership, and Christian religiousness were linked to increased organizational identification, which, in turn, was linked to the intensification of UPB. The correlation between state ownership of an enterprise and Christian religiousness was also positive. State ownership of an enterprise was linked to a lower intensity of UPB when the level of organizational identification was controlled.

In accordance with hypothesis 1, organizational identification influences UPB. The value of the influence of organizational identification on unethical pro-organizational behavior was 0.21 (see Fig 3). This fits within the range of 0.058÷0.268 obtained as the result of correlation between these two variables, which was calculated with bootstrapping (Table 7). Thus, hypothesis 1 was confirmed by our study findings. This is consistent with the proposal of Umphress et al. [9] and has been repeatedly confirmed by various authors [9, 23, 24].

Authentic leadership influenced organizational identification to a very significant extent. The value of the influence was 0.38 (Fig 3) and fit within the range of 0.306÷0.487 obtained as the result of correlation between these variables (Table 7). In turn, organizational identification influenced UPB at the level of 0.21, as described above. Hypothesis 2 was thus also confirmed by the study results. It is worth noting that authentic leadership, despite its powerful ethical component, does not exert a direct influence on UPB. It appears that the leadership theory in which the main virtue is faithfulness to oneself is perhaps not suitable for modeling ethically positive behavior and preventing employee engagement in UPB.

A study with the SEM method did not confirm hypothesis 3 (Fig 3.). The same was true for the result of correlation analysis (Table 7).

The results also demonstrate that Christian religion does not have a direct impact on diminishing UPB, although we assumed it would have a moderate impact. This result does not confirm the influence of an individual’s moral level on the decrease of their engagement in UPB, which was proposed by Umphress and Bingham [6].

Christian religiousness exerts a statistically significant influence on employees’ organizational identification (0.10) and–due to its intermediary role–on intensification of engagement in UPB (Fig 3). Hence, we have confirmed hypothesis 4. In our study, not only did religiousness not influence the decrease in engagement in UPB but it also intensified engagement in UPB through the intermediary role of organizational identification. It was surprising to find an intercorrelation between employees of SOEs and religiousness at the level of 0.13. According to research, more conservative people are eager to select SOEs as their workplace. One of the state-owned groups of companies from which the respondents were recruited was a mining-metallurgical company with a traditional work ethos, in which religion plays a part. This may have influenced the results.

In our study, the group of respondents from SOEs distinguished themselves by displaying lower engagement in UPB. Their influence reached the level of -0.17, as presented in Fig 3. Therefore, the results confirm hypothesis 5 put forward in our study, although this did not seem to be the case based on the earlier correlation analysis (Table 7). In all likelihood, the reason for this finding is that attitudes toward work and company goals differ from those of employees in private entities; that is, this finding arises from the fact that SOEs are hybrid companies [128]. We do not claim that employees of such enterprises behave more ethically at work. They are, however, less ready to undertake UPB than the other respondents.

Employees of SOEs are characterized by greater organizational identification than the other respondents (0.29 in Fig 3), and organizational identification influences UPB at the level of 0.21 (Fig 3). The above results confirm hypothesis 6 put forward in our study. Thus, decreased engagement in UPB does not nullify the positive influence of SOEs on organizational identification, which consequently intensifies employees’ UPB.

## Discussion

Notably, authentic leadership, despite its powerful ethical component, does not exert a direct influence on UPB. Similar findings were obtained in an earlier study in Poland [20], in which the respondents were mainly young workers below 29 years of age. Our findings are similar for a group composed mainly of equal numbers of employees between the ages of 30 and 39 and 40 and 49. In total, these employees constituted 50.9% of the respondents. In both cases, authentic leadership exerted influence on the increase in followers’ engagement in UPB through organizational identification. It appears that the leadership theory in which the main virtue is faithfulness to oneself is perhaps not suitable for modeling ethically positive behavior and preventing employee engagement in UPB [64]. This outcome demonstrates the insufficiency of ethical business concepts in the context of the common phenomenon of engaging in UPB.

Our study, therefore, links earlier studies that have not registered the influence of religion on business ethics. The fact that hypothesis 3 was not confirmed correlates with the articles that indicate the lack of influence of religiousness on ethical conduct at work [113–115]. Nevertheless, our study on employees of SOEs produced different results than the Chinese studies [16] that showed no differences between engagement in UPB among employees of SOEs and non-SOEs. However, the research sample in the study consisted of managers of a single MBA studies program.

### Theoretical and managerial implications

Earlier research often brought about results that demonstrated the influence of leadership on intensified UPB among the followers [13, 14, 18]. This sometimes happens directly, but more commonly takes place through the agency of other phenomena, including organizational identification [23, 24]. This raises doubts as to the ethical content of normative theories of leadership [20]. Earlier studies were concerned with the theory of ethical leadership. Our study has contributed to the increase of the volume of knowledge regarding the influence of authentic leadership on UPB. AL is a universal concept which is applicable to various types of organizations [38, 72].

It is worth noting that authentic leadership, despite its powerful ethical component, does not exert a direct influence on UPB. Similar findings were obtained in an earlier study in Poland [20]. In both cases, authentic leadership exerted influence on the increase in followers’ engagement in UPB through work engagement or through organizational identification. It appears that the leadership theory in which the main virtue is faithfulness to oneself is perhaps not suitable for modeling ethically positive behavior and preventing employee engagement in UPB [64]. This outcome demonstrates the insufficiency of ethical business concepts in the context of the common phenomenon of engaging in UPB.

In a study on the influence of individual morality on unethical pro-organizational behavior, we conducted pioneering research on the factors related to religion. We were surprised by the lack of correlation between religiousness and decreased engagement in UPB. Our study, therefore, links to earlier studies that have not registered the influence of religion on business ethics. The fact that hypothesis 3 was not confirmed correlates with the articles that indicate the lack of influence of religiousness on ethical conduct at work [113–115]. Our study has contributed to this direction of research, but it seems that it is worth examining the issue further.

Our study on employees of SOEs produced different results than the Chinese studies [16] that showed no differences between engagement in UPB among employees of SOEs and non-SOEs. However, the research sample in the study consisted of managers in a single MBA studies program. Our study was conducted from a completely different cultural and geographical perspective. Additionally, in our study, there was an unexpected correlation found between religiousness and state ownership of an enterprise, which can be explained by the fact that in Poland the state sector encompasses traditional industries with more conservative and more religious employees. This research direction should be pursued further in the future.

### Limitations and future research

There are limitations to our study. First, one should be cautious with regard to generalization of the findings because the respondents were not selected from randomly designated SOEs; instead, we drew respondents from large groups of companies (each of which had several or several dozen subsidiaries). Second, since there was only one round of the study, temporal precedence between organizational identification and UPB cannot be confirmed [186]. Third, the religiousness scale that we employed does not take into consideration the religious practices of the respondents, but rather their religious beliefs. Theoretically, a person who is not religious himself or herself could have a high score if for some reason he or she believes that the Christian religion should play a serious part in society. Furthermore, we did not study leaders and followers in dyads; we studied subordinates only. We do not know what attitudes toward UPB are displayed by superiors, which may affect the results. Finally, although earlier research and the relevant literature strongly confirm our research model, the possibility of reverse causation of the obtained results cannot be ruled out.

Future research could be concerned with a more precise definition of the influence of religion on UPB while taking into account the respondents’ religious beliefs, as well as their practices. In studies on the relationship between leadership and UPB, other leadership concepts must be addressed. Authentic leadership with ethics based on sincere insight into oneself and loyalty toward one’s own beliefs may be insufficient. It seems more promising to use the theory of servant leadership, which is based on the ethics of caring for all stakeholders, including clients and counterparties [64]. SOEs, as hybrid organizations, are also interesting entities to study with regard to UPB. It appears to be worth examining whether organizational identification exhibits different characteristics in SOEs versus in private companies. Organizational identification in SOEs itself should be more thoroughly examined. Perhaps this organizational commitment means that there is a sense of some kind of “ownership” of the enterprise, which might be a communist legacy and not necessarily consistent with the goals of the top management of the enterprise. These leads are worth following in future research.

## Conclusions

We have reinforced the thesis that authentic leadership–a popular theory in business and science–influences the increase in UPB. This carries implications for the development of leadership theory and the practical application of authentic leadership. In the study of the influence of individual morality on UPB, we took into account employees’ religiousness. While elaborating on the issue of the influence of corporate culture on UPB, we paid attention to SOEs. Finally, we presented the perspective of Central and Eastern Europe, which carries cognitive value in the context of the universality of the construct of UPB.

Based on moral identity theory, social identity theory and social exchange theory, we investigated how organizational identification mediates correlations among SOEs, authentic leadership, religiosity and UPB. Leadership can be a double-edged sword. On the one hand, it promotes organizational identification and work engagement. On the other hand, leadership may encourage UPB by employees. In the long run, this is detrimental to the company and its stakeholders if it is not balanced by ethical codes that are conscientiously implemented in companies.

## Supporting information

S1 Data (XLSX)
